# Placental Origin of Prostaglandin F_**2**α****_ in the Domestic Cat

**DOI:** 10.1155/2014/364787

**Published:** 2014-02-11

**Authors:** Marta J. Siemieniuch, Ewelina Jursza, Anna Z. Szóstek, Lina Zschockelt, Alois Boos, Mariusz P. Kowalewski

**Affiliations:** ^1^Department of Reproductive Immunology and Pathology, Institute of Animal Reproduction and Food Research of the Polish Academy of Sciences, Tuwima Street, Olsztyn 10-748, Poland; ^2^Leibniz Institute for Zoo and Wildlife Research, Alfred-Kowalke Straße 17, 10315 Berlin, Germany; ^3^Institute of Veterinary Anatomy, Vetsuisse Faculty, University of Zurich, Winterthurerstrasse 260, 8057 Zurich, Switzerland

## Abstract

In the present study, the question was addressed whether the feline placenta can synthesize prostaglandin F_2**α**_ (PGF_2**α**_). The PGFS protein was elevated, particularly at 2.5–3 weeks of pregnancy compared to 7-8 (*P* < 0.05) and 8.5–9 weeks (*P* < 0.001). Transcripts for PGFS were significantly upregulated at 2.5–3 weeks of pregnancy and then gradually declined towards the end of gestation (*P* < 0.001). Transcripts for PTGS2 were only upregulated in placentas from queens close to term (*P* < 0.001) compared with earlier phases. Staining of PTGS2 showed distinct positive signals in placentas obtained during the last week before labor, particularly in the strongly invading trophoblast surrounding blood vessels, and also in decidual cells. Shortly after implantation, signals for PGFS were localized in the trophoblast cells. Near term, PGFS staining was seen mainly in decidual cells. Both placental PGF_2**α**_ and plasma PGFM were elevated towards the end of pregnancy (*P* < 0.001) compared with earlier weeks of pregnancy. The content of PGF_2**α**_ in extracted placenta mirrored the PGFM level in plasma of pregnant females. During late gestation there is a significant increase in PGFM levels in maternal blood and of PGF_2**α**_ levels in placental tissue concomitant with an upregulation of placental PTGS2.

## 1. Introduction

The domestic cat (*Felis catus*) is seasonally polyestrous, with ovulation usually provoked by coitus [1, 2]; however in as much as 30–50% of the cycles, ovulation is spontaneous [3]. During the breeding season, which extends from February-March until late summer in moderate latitudes [4], the cat exhibits several estrous cycles. If ovulation is not followed by pregnancy, the queen enters pseudopregnancy (a nonpregnant luteal phase) that lasts only about one-half of a normal gestation [5]. The length of the luteal phase in pregnant queens, accompanied by elevated progesterone (P_4_) levels, is assumed to be partially driven by luteotrophic and luteolytic factors of placental origin [6]. In the dog, the uteroplacental unit seems to serve as the major source for the prepartum release of the luteolytic PGF_2*α*_ [7–9].

Even though the luteal phase in pseudopregnant cats is much shorter than in the dog, one may speculate that the feline CL possesses an intraluteal mechanism that triggers its demise at the appropriate time. The life-span of the CL in the pregnant cat is longer than in pseudopregnant queens; however, it needs to be precisely regulated to allow the onset of parturition. Although PGF_2*α*_ seems to be a very potent luteolysin, its role in regulating the CL of pregnancy in cats is still uncertain. Shille and Stabenfeldt [10] showed that high doses and repeated treatments with PGF_2*α*_ on days 11–15 after mating had almost negligible effects on progesterone levels in cats, which initially diminished but shortly afterwards recovered completely following treatment. Similarly, PGF_2*α*_ given on days 4 and 5 or 12 and 13 had no effect nor on circulating P_4_ neither on CL size as monitored laparoscopically [11]. In female cats on days 21–25 after mating, luteal function was transiently depressed after treatment, but eventually the treatment did not alter the length of the luteal phase compared with untreated cats [10]. However, exogenously administered PGF_2*α*_ or its analogue has a luteolytic effect on fully developed CLs after day 33 [12] or day 40 of gestation [13].

Recently, the PGF_2*α*_ inactive metabolite 13,14-dihydro-15-keto prostaglandin F_2*α*_ (PGFM), measured in the feces, was shown to be a precise pregnancy indicator in several felid species, including the domestic cat, during the last trimester of gestation [14]. The uteroplacental complex was proposed to be a source of PGF_2*α*_ in cats [14]. We hypothesize that the placenta of the domestic cat synthesizes PGF_2*α*_ in a time-dependent manner. To address this hypothesis, we examined in the feline placenta (1) the cellular localization of PGFS (AKR1C3), (2) the PGFS protein and mRNA content, (3) the cellular localization of PTGS2 and its mRNA expression, and (4) the tissue level of PGF_2*α*_ at different gestational stages. Moreover, in maternal blood plasma the PGF_2*α*_ metabolite PGFM was measured throughout gestation.

## 2. Materials and Methods

### 2.1. Animals, Tissue Sampling, and Preservation

All procedures were approved by the Local Animal Care and Use Committee in Olsztyn, Poland (61/2010/DTN). Thirty domestic, healthy cats, aged 1–6 years were brought into a local veterinary clinic for neutering. Ovariohysterectomy was done with the owner's request and consent. Four groups were created with queens ovariohysterectomized according to the following schedule.

1. Postimplantation, early gestation, days 18–21 (2.5–3 weeks), *n* = 8.
2. Mid gestation, days 28–35 (4-5 weeks), *n* = 8.
3. Late gestation, days 49–56 (7-8 weeks), *n* = 7.
4. Before parturition, days 60–64 (8.5–9 weeks), *n* = 6.

Additionally, samples were collected from one cat during hysterectomy around day 14 of gestation (peri-implantation period).

The day of mating, known in 18 females, was recorded as day 0. Confirmation of gestational age, whenever the day of mating was unknown, was done according to the measurements of the crown-rump length in fetuses and uterine ampullae diameters or lengths [15, 16]. In addition, measurements of the progesterone (P_4_) values reported in the literature [17] and confirmed in our last report [18] were performed. Blood was drawn preanesthesia. Blood samples were collected from the cephalic vein into EDTA-containing tubes (Tyco Healthcare Group LP, Mansfield, USA) and transported to the laboratory at 4°C. Plasma obtained after blood centrifugation (3500 ×g, 10 min) was frozen at −20°C until P_4_ and PGFM measurements . Direct enzyme immunoassay (EIA) was done for hormonal analysis.

Tissues were washed immediately after surgery with sterile saline to remove blood contamination then were placed into fresh sterile saline at 4°C and transported to the laboratory within 1 h. Uterine horns were slit longitudinally and pieces of uterus with attached placenta were prepared, washed in fresh saline to remove blood, preserved overnight at 4°C with RNAlater (Ambion Biotechnologie GmbH, Wiesbaden, Germany), and then stored at −80°C until total-RNA extraction. Another fragment of the same placenta was fixed in buffered 4% formaldehyde for 24 h, dehydrated, and wax-embedded. Sections (2-3 *μ*m thick) were used to determine PGFS and PTGS2 protein localization by immunohistochemistry. Transcripts of PGFS and PTGS2 were determined using Real-Time PCR, while the content of PGFS protein was determined by western blot analysis.

### 2.2. RNA Isolation, Reverse Transcription, and Real-Time PCR

Total RNA was isolated from feline placenta using TRIZOL-reagent according to the manufacturer's instructions (Sigma Aldrich, Warszawa, Poland). The RNA content was measured with a Nanodrop 1000 Spectrophotometer (Thermo, USA). Prior to reverse transcription, genomic DNA contamination was removed by treatment with DNase (Sigma Aldrich, Warszawa, Poland). Reverse transcription was performed using the ImProm-II Reverse Transcription System (Promega, Warszawa, Poland) according to the manufacturer's protocol. In brief, an experimental reaction contained 1 *μ*g of RNA in 12 *μ*L of reaction mix, 4 *μ*L of reaction buffer, 2.5 *μ*L of MgCl_2_ (final concentration 3 mM), 1 *μ*L of dNTP Mix (final concentration 0.5 mM each dNTP), and 1 *μ*L of ImProm-II Reverse Transcriptase. The final volume of the RT reaction mix was 20.5 *μ*L. The tubes were placed in a thermocycler (Bio-Rad Laboratories, Hercules, CA, USA) equilibrated at 25°C and incubated for 5 min. For the next step, tubes were incubated in a thermocycler for 1 h at 42°C. For Reverse Transcriptase inactivation, the temperature was increased to 70°C for 15 min. Samples were kept at −20°C until analyzed by Real-Time PCR.

The levels of mRNA expression of target genes were examined by Real-Time PCR using specific primers for *PGFS*, *PTGS2, *and *cyclophilin A (Cyc)*. Among several housekeeping genes that have been already examined for their stability and efficiency [Jursza, Siemieniuch, 2013, unpublished], *Cyc* was used as a housekeeping gene. All primers were purchased from Sigma Aldrich (Warszawa, Poland) and were tested in our previous report [18, 19]. The sequences were as follows: *PGFS* forward: 5′-TCAACCAGAGCAAACTGCTG-3′, *PGFS* reverse: 5′-CATTCCTTCCCTGAGTTGGA-3′ (GenBank accession number HM490147); *PTGS2* forward: 5′-AACAGGAGCATCCAGAATGG-3′; *PTGS2* reverse: 5′-GCAGCTCTGGGTCAAACTTC-3′ (GenBank accession number EF036473); *Cyc* forward: 5′-CCTTCTGTAGCTCGGGTGAG-3′; *Cyc* reverse: 5′-CTTGGAGGGGAGGTAAGGAG-3′ (GenBank accession number AY029366).

The Real-Time PCR reactions were carried out in an automated fluorometer ABI PRISM 7300 Sequence Detection System (Applied Biosystems, Darmstadt, Germany) using SYBR Green Master Mix (Applied Biosystems, Applera, Warsaw, Poland). The PCR reactions were performed in 96-well plates. The total reaction volume was 20 *μ*L containing 1 *μ*L cDNA (200 ng), 500 nM each of forward and reverse primers, and 10 *μ*L SYBR Green PCR Master Mix. Real-time PCR was carried out as follows: initial denaturation (10 min at 95°C), followed by 40 cycles of denaturation (15 s at 95°C), and annealing (1 min at 60°C). After each PCR reaction, melting curves were obtained by stepwise increases in temperature from 60 to 95°C to ensure single product amplification. The presence of the product was also confirmed by electrophoresis on 2% agarose gel. Relative quantification was performed by normalizing the signals of target genes with the *Cyc* signal using the Miner method for quantifying qRT-PCR results, using calculations based on the kinetics of individual PCR reactions [20].

### 2.3. Protein Preparation and Western Blotting

Tissue homogenates were prepared with NET-2 lysis buffer containing 50 mM Tris-HCl, pH = 7.4, 300 mM NaCl, 0.05% NP-40, and protease inhibitor cocktail (10 *μ*L/mL). Tissues were centrifuged at 10,000 g for 10 min at 4°C; then proteins in the supernatant were disrupted using a sonicator (75 W for 15 s). The concentrations of protein were determined by the Bradford assay with a spectrophotometer (SmartSpec Plus, Bio-Rad). Proteins were solubilized in sample buffer (25 mmol/L Tris-Cl, pH = 6.8, 1% SDS, 5% *β*-mercaptoethanol, 10% glycerol, and 0.01% bromophenol blue), placed onto 12% SDS-polyacrylamide gel, and separated at 120 V. Afterwards, proteins were transferred onto methanol-activated PVDF membranes for 1 h at 100 V. To block nonspecific binding, membranes were incubated for 1 h in nonfat dry milk, 5% in PBS with 0.25% Tween 20. Membranes were incubated overnight at 4°C with canine-specific guinea pig polyclonal affinity purified anti-PGFS (AKR1C3) custom-made antibody (Eurogentec S.A., Seraing, Belgium), at a dilution of 1 : 400, as described by Gram et al. [8]. After this, membranes were washed three times for 10 min in PBS with Tween-20 at 20°C. Next, membranes were incubated with horseradish peroxidase (HRP)-conjugated secondary antibody (Sigma, dilution 1 : 15.000) for 1 h at room temperature (RT).

As a loading control, PVDF were reblotted with anti-*β*-actin mouse monoclonal antibody (sc-69879, Santa Cruz Biotechnology, Rockford, IL, USA, dilution 1 : 1000). Results were visualized using the SuperSignal West Femto Maximum Sensitivity Substrate according to the manufacturer's protocol (Thermo Scientific, USA) and the ChemiDoc XRS+ System and Image Lab (BioRad). The optical density of bands was determined using ImageJ software.

### 2.4. Immunohistochemistry

Formalin-fixed, paraffin-embedded tissues were cut with a microtome (2-3 *μ*m) and mounted on SuperFrost Plus microscope slides (Menzel-Gläser, Braunschweig, Germany). The experimental protocol was as previously described for feline placenta [18]. Briefly, slides were deparaffinized and rehydrated in a graded ethanol series and then incubated in citrate buffer (10 nM, pH 6.0) for 15 min under microwave irradiation at 560 W for antigen retrieval. Then, sections were incubated in 0.3% H_2_O_2_ in methanol for 30 min to quench endogenous peroxidase and then washed in IHC-buffer/0.3% Triton X pH 7.2–7.4 (0.8 mM Na_2_HPO_4_, 1.47 mM KH_2_PO_4_, 2.68 mM KCl, and 1.37 mM NaCl). Blocking of nonspecific binding sites was performed in 10% goat or rabbit serum. The following primary antibodies were used: canine-specific guinea pig polyclonal affinity purified anti-PGFS (AKR1C3) antibody (dilution 1 : 400) (Eurogentec S.A., Seraing, Belgium), the same as for western blot and as recently applied for canine uteroplacental tissue [8], and affinity purified goat anti-rat polyclonal antibody raised against the N-terminus of COX2 (Santa Cruz Biotechnology, Inc., CA, USA). The negative controls were as follows: slides without the primary antibody and slides incubated with nonimmunized guinea pig or goat serum, respectively, at the same dilution and protein concentration as primary antibodies. Sections were incubated overnight at 4°C. After washing with IHC-buffer, slides were incubated for 30 min at RT with either biotinylated goat IgG (secondary antibody, dilution 1 : 100) against guinea pig immunoglobulin (Vector Laboratories, Burlingame, US) or biotinylated rabbit IgG antibody (dilution 1 : 100) against goat immunoglobulin (Vectastain ABC Kit, Vector Laboratories). For enhancing signals, sections were incubated with the avidin-biotin-peroxidase complex (Vectastain ABC kit, Vector Laboratories) for 30 min at RT. After washing with IHC-buffer, sections were allowed to react with the substrate diamino-benzidine (DAB; DakoCytomation, Glostrup, Denmark) according to the manufacturer's instructions. Slides were counterstained with hematoxylin, rinsed under running tap water for 5 min, dehydrated in a graded ethanol series, and mounted in mounting medium DPX (Panreac Quimica Sau, Barcelona, Spain).

### 2.5. Placental PGF_2*α*_ Extraction

For PGF_2*α*_ extraction, the protocol elaborated by Tsang et al. [21] and tested for feline placental progesterone extraction by Siemieniuch et al. [18] was applied, with some minor modifications. Briefly, placental fragments weighing 200–220 mg were stored at −80°C. After thawing, each tissue sample was homogenized in a glass vial using a tissue disruptor with 400 *μ*L of a Tris buffered saline containing proteins and sodium azide as preservative and acidified by addition of 45 *μ*L 1 N HCl. After adding 3 mL of ethyl ether to the samples, they were vortexed for 10 min and incubated at −20°C for 4 h. Afterwards, the supernatant was collected and evaporated to dryness under a stream of nitrogen at 40°C. Finally, 400 *μ*L of the Tris buffered saline was added, mixed, and allowed to sit for 15 min at 20°C. The samples were stored at −20°C until the immunoassay was run.

### 2.6. Prostaglandin F_2*α*_ (PGF_2*α*_), 13,14-Dihydro-15-keto Prostaglandin F_2*α*_ (PGFM), and Progesterone (P_4_) Determination

For PGF_2*α*_ and PGFM measurements, the commercial PGF_2*α*_ high sensitivity EIA kit (ENZO Life Sciences Inc., Farmingdale, NY, USA) and 13,14-dihydro-15-keto Prostaglandin F_2*α*_ EIA kit (Cayman Chemical Company, Ann Arbor, MI, USA) were used, respectively, and run according to the manufacturers' instructions.

The sensitivity of the PGF_2*α*_ assay was 0.98 pg/mL. This assay is based on the competition between free PGF_2*α*_ and a PGF_2*α*_ tracer for a limited number of PGF_2*α*_-specific sheep antiserum binding sites. The cross-reactivity for various prostaglandins and their metabolites was as follows: PGF_2*α*_ 100%, PGF_1*α*_ 11.82%, PGD_2_ 3.62%, 6-keto-PGF_1*α*_ 1.38%, PGI_2_ 1.25%, and PGE_2_ 0.77%. The inter- and intraassay precision variation was 6.8% and 10.1%, respectively.

The sensitivity of the PGFM assay was 15 pg/mL. This assay is based on the competition between free 13,14-dihydro-15-keto PGF_2*α*_ and a 13,14-dihydro-15-keto Prostaglandin F_2*α*_ tracer for a limited number of 13,14-dihydro-15-keto PGF_2*α*_-specific rabbit antiserum binding sites. The cross-reactivity for various prostaglandins and their metabolites was as follows: 13,14-dihydro-15-keto PGF_2*α*_ 100%, 13,14-dihydro-15-keto PGE_2_ 2.7%, and 15-keto PGF_2*α*_ 1.8%. The inter- and intraassay precision variation was 8.3% and 14.5%, respectively.

For P_4_ measurement, a commercial Progesterone ELISA kit (ENZO Life Science) was used and run in accordance with the manufacturer's instructions. This kit uses a polyclonal antibody to P_4_ to bind, in a competitive manner, P_4_ in a sample or P_4_ with a covalently attached alkaline phosphatase molecule. The sensitivity of this P_4_ assay was 8.57 pg/mL. The cross-reactivity for a number of related steroids was as follows: P_4_ 100%, 5*α*-Pregnane-3,20-dione 100%, 10-OH-progesterone 3.46%, corticosterone 0.77%, and deoxycorticosterone 0.28%. The inter- and intraassay precision variation was 8.1% and 9.5%, respectively.

### 2.7. Statistics

To test the effect of different stages of pregnancy on mRNA levels and on PGFM or PGF_2*α*_ concentrations, the Kruskal-Wallis Test (a nonparametric ANOVA) was used followed by the Newman-Keuls Multiple Comparison Test using the statistical software program GraphPad6 (GraphPad PRISM v 6.0; GraphPad Software Inc., San Diego, CA, USA). PGFM concentrations in plasma or PGF_2*α*_ concentrations in the placenta are shown as a mean ± standard deviation. Significance was defined as values of *P* < 0.05.

## 3. Results

### 3.1. Placental Expression of PGFS throughout Pregnancy

**Figure 1:**
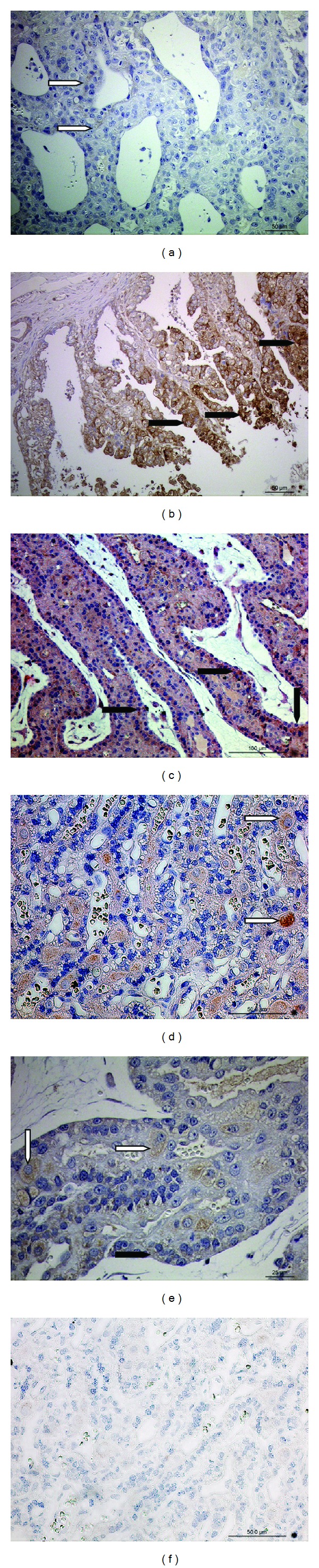
PGFS (AKR1C3) protein localization in the uteroplacental unit. Immunohistochemical (IHC) localization of PGFS in the week 2 of pregnant uterus (a) and placenta at week 3 (b), week 6 (c), and week 9 of gestation ((d), (e)). (a) In the feline uterus some weak PGFS signals are localized in the glandular epithelium (open arrowheads); ((b), (c)) in the placental labyrinth, PGFS expression is localized in the fetal trophoblast cells (solid arrowheads). ((d), (e)) In the fully developed placenta, towards the end of pregnancy, signals are weaker in trophoblast cells and are mostly localized in maternal decidual cells (open arrowheads). (f) Isotype control.

In the uterus at the peri-implantation period (2nd week of pregnancy), no or very weak signals were observed in the glandular epithelium (Figure 1(a)). After implantation (3rd week of pregnancy), and in fully developed placenta at the 7th week of gestation, strong placental signals were localized in fetal trophoblast cells (Figures 1(b) and 1(c), resp.). Towards the end of pregnancy (9th week) signals in trophoblast cells were much weaker, and were localized in the maternal decidual cells (Figures 1(d) and 1(e)). Negative controls showed no staining (Figure 1(f)).

**Figure 2:**
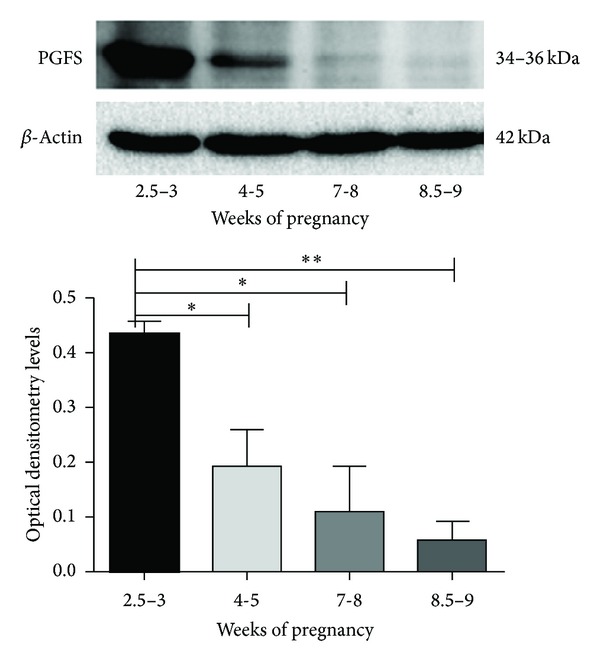
PGFS (AKR1C3) protein expression. Time-dependent expression of PGFS protein in the feline placenta. Tissue homogenates (30 *μ*g) were used in western blot analysis (*n* = 16). Representative immunoblots are shown. Lower panel represents densitometric values for PGFS normalized against B-actin (mean ± S.D.).

In the western blot analysis, the PGFS was detected as a protein with a molecular size of approximately 34–36 kDa. The expression of PGFS protein was strongly affected by gestational age (Figure 2). The PGFS protein was upregulated, particularly in the first period studied, at 2.5–3 weeks of pregnancy, compared to samples collected at 7-8 gestational weeks (*P* < 0.05) or 8.5–9 weeks (*P* < 0.001).

**Figure 3:**
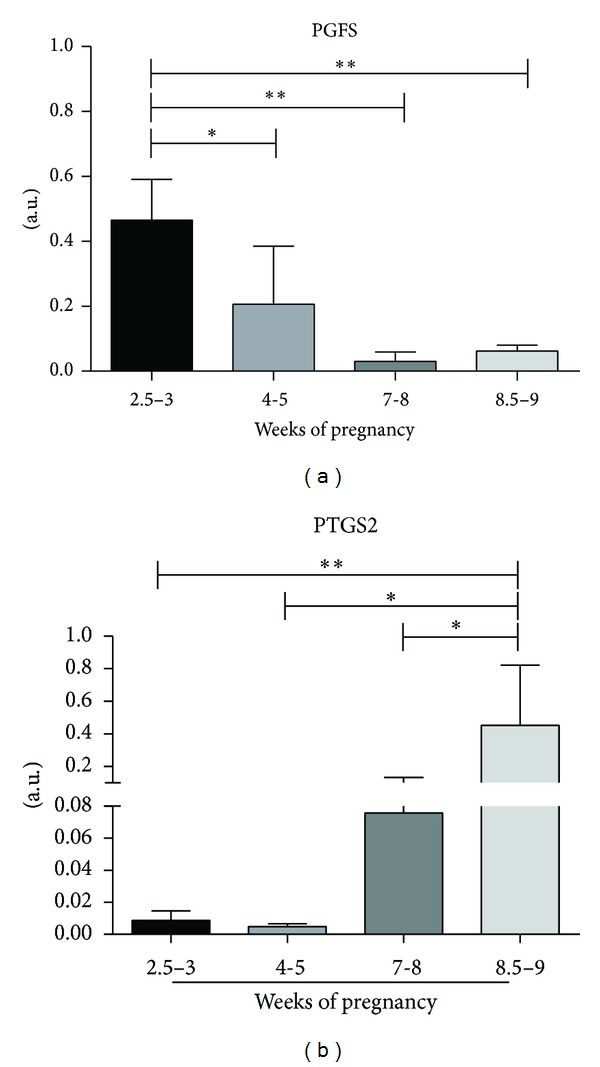
PGFS (AKR1C3) and PTGS2 mRNA expression. Time-dependent expression of feline PGFS (a) and PTGS2 (b) as determined by Real-Time PCR. Asterisks indicate statistical differences between expression levels during the course of pregnancy (*P < 0.05, **P < 0.001).

The expression of transcripts for placental PGFS as determined by Real-Time PCR was strongly time-dependent (Figure 3(a)). The PGFS mRNA transcripts were mirrored by PGFS protein expression in western blot analysis. The PGFS mRNA was significantly upregulated at the 3rd week of pregnancy and then gradually declined towards the end of gestation (*P* < 0.001).

### 3.2. Placental Expression of PTGS2 throughout Pregnancy

**Figure 4:**
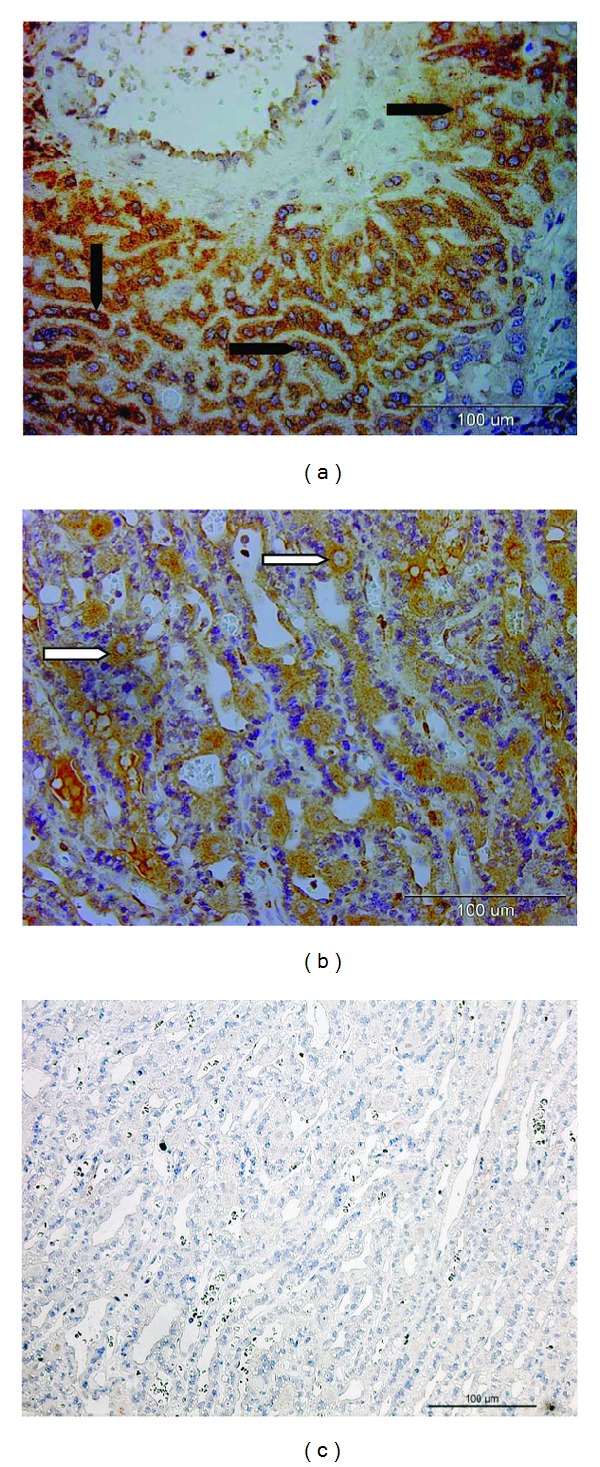
PTGS2 protein localization in the placenta. Immunohistochemical localization of PTGS2 in feline placenta from the 9th week of pregnancy. Positive signals are shown in the strongly invading trophoblast surrounding blood vessels (solid arrowheads) (a) and also in maternal decidual cells (open arrowheads) (b). (c) Isotype control.

The IHC analysis revealed abundant positive signals in 9th week feline placenta. Strong staining was observed in the invading fetal trophoblast cells surrounding maternal blood vessels (Figure 4(a)). Similarly, strongly positive signals were observed in the maternal decidual cells in the 9th week of pregnancy (Figure 4(b)). No signals were noted in the negative controls (Figure 4(c)). Only weak signals were observed for placental PTGS2 protein expression during earlier stages of pregnancy.

The expression of transcripts for placental PTGS2, as determined by Real-Time PCR, was time-dependent (Figure 3(b)). PTGS2 mRNA expression was basal during early and midgestation, started to increase during late gestation, and was significantly upregulated during the prepartal phase (*P* < 0.001).

### 3.3. Placental Prostaglandin F_2*α*_ (PGF_2*α*_) and Blood Plasma 13,14-Dihydro-15-keto Prostaglandin F_2*α*_ (PGFM) Content

**Figure 5:**
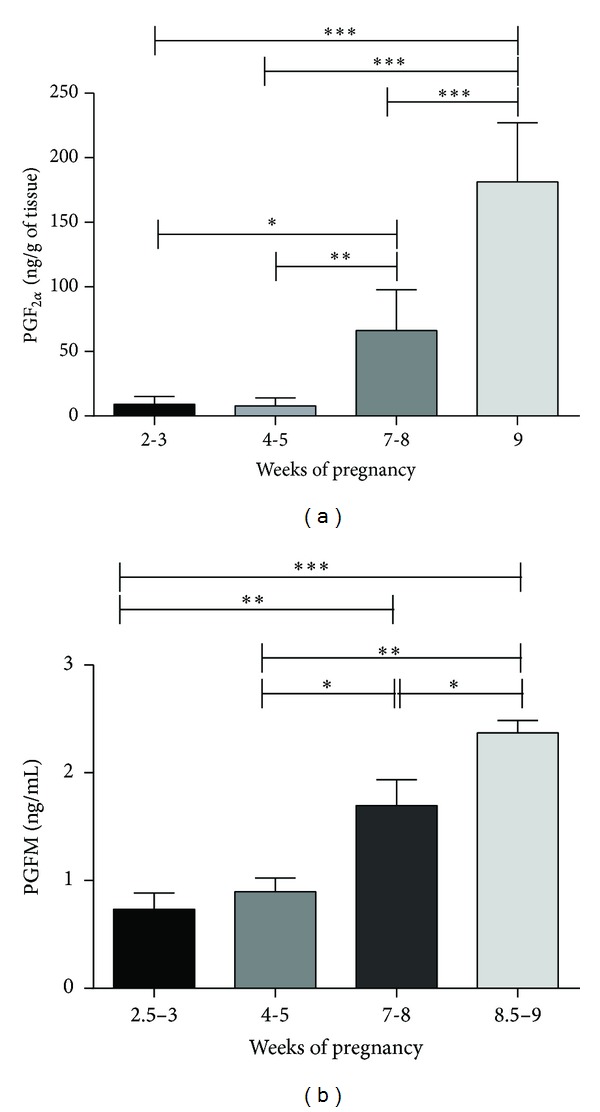
Placental PGF_2*α*_ and serum PGFM values. Time-dependent content of PGF_2*α*_ in placenta (a) and PGFM in blood plasma (b) as determined by immunoassays. Asterisks indicate statistical differences between prostaglandin levels during the course of pregnancy (*P < 0.05, **P < 0.001, and ***P < 0.0001).

The levels of PGF_2*α*_ extracted from placentas mirrored the PGFM levels measured in peripheral blood plasma of pregnant females throughout gestation, as shown in Figures 5(a) and 5(b), respectively. Both placental PGF_2*α*_ and plasma PGFM were elevated towards the end of pregnancy compared to the values obtained at the 3rd week of pregnancy (*P* < 0.001).

## 4. Discussion

To examine the role of prostaglandins in the feline placenta near the end of pregnancy, the transcripts for PTGS2 and its cellular localization were analyzed. PTGS2, the crucial enzyme in generating prostaglandin, catalyzes the conversion of arachidonic acid into endoperoxide PGH_2_, which is a precursor of all prostaglandins [22, 23]. In the present study, transcripts for PTGS2 were upregulated in placentas solely from queens in the last week before termination of the pregnancy. These findings are in agreement with a previous report of an acute increase of PTGS2 transcripts in the placenta in the dog during prepartum luteolysis [7]. In the present study, the protein localization of PTGS2 was found in both fetal trophoblast and maternal decidual cells. In contrast, both protein and transcripts for PGFS (AKR1C3) were strongly upregulated soon after implantation, which takes place on days 12–14 after ovulation in the cat [24]. Transcripts for PGFS and PGFS protein were rather low in the placenta near the end of pregnancy. These findings are in agreement with a report of decreased PGFS transcripts in preparturient dogs, which led to the conclusion that PGFS (AKR1C3) might be not responsible for the sharp PGF_2*α*_ elevation just before term in the bitch [7].

One of the questions addressed in this study was whether PGFS (AKR1C3), that was shown to promote the direct conversion of PGH_2_ to PGF_2*α*_ [8], is responsible for placental elevation of PGF_2*α*_. In the dog, PGFS (AKR1C3) staining was predominantly localized in the fetal trophoblast cells in the fetomaternal contact zone, justifying the assumption that PGFS may play a role in decidualization and placentation; moreover, PGFS expression was low prior to implantation but distinctly increased thereafter [8]. The cellular localization of PGFS in the feline placenta from 3 weeks of gestation is similar to that observed in the dog. Therefore, PGFS is implicated in the processes of fetal membrane development and placentation. The discrepancy observed between elevated PGF_2*α*_ concentration in the placenta and downregulation of PGFS (AKR1C3) protein and mRNA levels may be explained by the number of enzymes involved in generation of the wide variety of prostaglandins. PGF_2*α*_ is one of the few primary prostaglandins derived enzymatically from the endoperoxide PGH_2_. It can be directly synthesized from PGH_2_ by PGF_2*α*_ synthase (PGFS), like AKR1C3 presented herein. However, other pathways of PGF_2*α*_ synthesis, including enzymes belonging to the aldo-ketoreductases family, for example, AKR1B1 or AKR1B5, are possible. The canine PGFS cDNA sequence has been cloned [25] and classified as an AKR1C3 and is the only canine-specific PGFS isoform known so far [8]. The feline PGFS cDNA sequencing was based on a dog cDNA sequence and showed 87% homology with the canine PGFS [19]. Furthermore, PGF_2*α*_ may originate from conversion of several other prostaglandins, including catalysis of PGE_2_ to PGF_2*α*_ by 9-keto-reductase (9KPGR), or perhaps even at relatively low expression levels AKR1C3 is still not rate-limiting for PGF_2*α*_ synthesis.

Recently, elevated concentrations of a stable PGF_2*α*_ metabolite (PGFM) were found in the feces of several felid species in the last trimester of pregnancy [14]. In the present study, the PGFM level, measured in feline plasma blood collected just before ovariohysterectomy, was low during the first and second trimesters of pregnancy and then started to increase for the last 3 weeks of gestation. Certainly, PGFM in the maternal plasma may not originate exclusively from the placenta. Nevertheless, to examine whether the placenta might support synthesis of PGF_2*α*_, placental tissues from different gestational weeks were extracted to obtain PGF_2*α*_. The PGFM blood plasma level was mirrored by the placental PGF_2*α*_ concentration. The lowest amount of PGF_2*α*_ was observed in tissue collected soon after implantation, indicating there is no likely biological role at this time. It explains why in the earlier studies by Wildt and coworkers [11] or Shille and Stabenfeldt [10] PGF_2*α*_ failed to influence P_4_ production or reduce CL size. The first rise in concentration was seen at the beginning of the second trimester, but the most distinct increase coincided with the last week of gestation. The very similar pattern of placental PGF_2*α*_ and its stable metabolite in feline blood plasma may suggest the placental origin of PGFM. However, this finding does not rule out an intraluteal and/or uterine source of PGF_2*α*_ during pregnancy, especially since components of the underlying synthetic pathways were already found in the domestic cat CL (Zschockelt and Siemieniuch 2013, unpublished). This is in contrast with the canine CL, in which intraluteal prostaglandin synthesis is associated with luteal formation but not with luteal regression or luteolysis [8, 26, 27]. The strong prepartum increase in circulating PGF_2*α*_ observed in the dog seems to be originated from the uteroplacental compartment, where strongly upregulated expression of the prostaglandin system was reported [7] and was evidenced by the increased output of PGF_2*α*_ from the canine placenta at the time of parturition [9]. Furthermore, the prepartum increase in peripheral PGF_2*α*_ in the dog is accompanied by a steep P_4_ decline, implicating its role during luteolysis and/or fetal expulsion [28, 29]. These results partially agree with our present observations concerning elevated PGFM levels in maternal blood in the domestic cat and are consistent with the previous report from our laboratory presenting a considerable decline of P_4_ in both maternal plasma and placental tissue in the last week of feline pregnancy [18]. The latter observation, however, concerning the P_4_ levels does not relate to the immediate prepartum time period but describes the strongly diminished P_4_ levels during the last gestational week [18]. At least, the luteolytic role of PGF_2*α*_ in the second half of feline gestation is generally accepted, since PGF_2*α*_ or its analogues given to females with fully developed CLs induces luteolysis and consequent abortion after day 33 [12] or day 40 of gestation [13].

In conclusion, the data presented here confirms that the placenta of the domestic cat is capable of synthesizing PGF_2*α*_ in a time-dependent manner. Besides the fact that AKR1C3 is not a rate limiting enzyme even at relatively low expression levels, it may still be determinant for PGF_2*α*_ synthesis in the feline placenta. However, other enzymes and other synthetic pathways must also be taken into account. The peak of both placental PGF_2*α*_ and maternal plasma PGFM coincides with the beginning of the last week of pregnancy in cats, compared to dogs, in which peak plasma PGFM is observed 24–48 h before term. Other pathways involved in PGF_2*α*_ synthesis in the cat placenta are possible and need to be elucidated.
